# Proteomics in Inherited Metabolic Disorders

**DOI:** 10.3390/ijms232314744

**Published:** 2022-11-25

**Authors:** Maria del Pilar Chantada-Vázquez, Susana B. Bravo, Sofía Barbosa-Gouveia, José V. Alvarez, María L. Couce

**Affiliations:** 1 Proteomic Platform, Health Research Institute of Santiago de Compostela (IDIS), Hospital Clínico Universitario de Santiago de Compostela, 15706 Santiago de Compostela, Spain; 2 Department of Forensic Sciences, Pathology, Gynecology and Obstetrics, Pediatrics, Neonatology Service, Department of Pediatrics, Hospital Clínico Universitario de Santiago de Compostela, Health Research Institute of Santiago de Compostela (IDIS), CIBERER, MetabERN, 15706 Santiago de Compostela, Spain

**Keywords:** biomarkers, enzyme replacement therapy, inborn errors of metabolism, lysosomal disorders, proteomics

## Abstract

Inherited metabolic disorders (IMD) are rare medical conditions caused by genetic defects that interfere with the body’s metabolism. The clinical phenotype is highly variable and can present at any age, although it more often manifests in childhood. The number of treatable IMDs has increased in recent years, making early diagnosis and a better understanding of the natural history of the disease more important than ever. In this review, we discuss the main challenges faced in applying proteomics to the study of IMDs, and the key advances achieved in this field using tandem mass spectrometry (MS/MS). This technology enables the analysis of large numbers of proteins in different body fluids (serum, plasma, urine, saliva, tears) with a single analysis of each sample, and can even be applied to dried samples. MS/MS has thus emerged as the tool of choice for proteome characterization and has provided new insights into many diseases and biological systems. In the last 10 years, sequential window acquisition of all theoretical fragmentation spectra mass spectrometry (SWATH-MS) has emerged as an accurate, high-resolution technique for the identification and quantification of proteins differentially expressed between healthy controls and IMD patients. Proteomics is a particularly promising approach to help obtain more information on rare genetic diseases, including identification of biomarkers to aid early diagnosis and better understanding of the underlying pathophysiology to guide the development of new therapies. Here, we summarize new and emerging proteomic technologies and discuss current uses and limitations of this approach to identify and quantify proteins. Moreover, we describe the use of proteomics to identify the mechanisms regulating complex IMD phenotypes; an area of research essential to better understand these rare disorders and many other human diseases.

## 1. Introduction

There are more than 7000 known rare diseases, and more are discovered each year [1]. Although individually rare, their global prevalence is high: they affect approximately 300 million people worldwide and often cause chronic illness, disability, and early death. Moreover, a large number of rare disease patients remain undiagnosed for many years and many even die without receiving an accurate diagnosis of their disease.

Over 80% of rare diseases have a genetic origin [1]. In recent years, genetic testing using next generation sequencing (NGS), that allows whole exome and whole genome sequencing, has helped to uncover the molecular basis of some rare and undiagnosed diseases. However, these technologies have enabled diagnosis for only 25–35% of previously undiagnosed patients [1,2]. Moreover, functional studies cannot always identify the cellular basis or molecular implications of these mutations, underscoring the need for further novel technological approaches. The development of effective treatments for rare diseases represents another major challenge. To date, treatments are available for only around 5% of rare diseases.

Inherited metabolic disorders (IMD) are rare genetic conditions; their diagnosis and treatment is based on biomarker-driven approaches. Genes, proteins, and metabolites are the most frequently used biomarkers. The IEM base database (http://www.iembase.org (accessed on 25 September 2022) currently contains about 1450 inborn errors of metabolism (IEM) [3], accounting for nearly 20% of all rare diseases, and incidence rates are 1 in 800–2500 live births [4,5]. From a pathophysiological point of view, IMDs can be divided into three diagnostically useful groups: (i) defects of small molecules; (ii) disorders involving energy metabolism; and (iii) disorders involving complex molecules [6]. IMDs can present with very varied symptoms and signs, affecting any organ at any stage of life between the prenatal period and adulthood. Some present acutely, while others have more indolent symptoms, affecting a number of organs; the central nervous system is one of the more commonly affected systems [7,8]. Although 88% of IMDs can be detected through biochemical tests of blood, urine, feces, fibroblasts, or cerebrospinal fluid, appropriate genetic laboratory tests are essential for accurate diagnoses [9,10].

The genetic bases of IEMs are very heterogeneous, and can involve genomic rearrangements, deletions or insertions, or one or more point mutations, all of which induce loss or gain of function of mutant proteins (usually enzymes or transporters), with consequent effects on cellular flux.

IEMs are linked to genetic defects; therefore, the most frequently used approach for disease characterization is identification of the mutated gene and its products. However, for a given mutation, genotype does not always correlate with phenotype, making it difficult to predict the impact of this disruption on the patient. Therefore, it is crucial to integrate all available scientific skills and methods, including -omics techniques, in order to assess the altered cellular pathways and potential interactions with other pathways.

Here, we review the most recent tandem mass spectrometry (MS/MS)-based proteomic strategies used to study IMDs. Omics strategies will undoubtedly play an important role in the post-genomics era of health care. In fact, they are already being used to search for biomarkers in body fluid samples for many diseases (e.g., cancer, stroke, diabetes, obesity). Mass spectrometry (MS), which enables rapid and cost-effective evaluation of a broad range of metabolites from small samples, including dried blood spots (DBS), is currently being used to search for diagnostically useful metabolites in body fluids in patients with rare diseases [2]. The use of MS to analyze proteins will be one of the next steps in the search for diagnostic techniques and therapies for rare diseases.

## 2. Proteomic Strategies

The evolution of the “omics” technologies has provided useful methods for in-depth, unbiased study of biological systems, and has led to the development of more precise diagnostic tools and therapies. With advancements in proteomics technologies, it is possible to simultaneously perform protein identification and quantification of expression profiles for thousands of proteins. Proteomic analysis has become a powerful method for characterizing the molecular profiles of patients, allowing analysis of both tissues [11] and biological fluids [12].

It is important to mention that organ-specific proteins released from diseased tissue are often diluted or degraded once they enter systemic circulation. Furthermore, serum contains a large proportion of proteins, such as albumin, with a wide dynamic range of abundance, making it difficult to reliably quantify these organ-specific biomarkers. Changes in serum protein levels may reflect alterations in inflammation that do not provide information about the disease process [13]. Therefore, an organ-specific disease sampling of fluid compartments near the diseased tissue (e.g., synovial fluid, urine, cerebrospinal fluid) can provide a more reliable source of potential diagnostic and therapeutic biomarkers than serum [11,12,13].

The emerging concept of personalized medicine relies on global and integrative approaches (such as proteomic technologies) to provide more precise patient care, and these technologies can also provide valuable information about the diseases [14,15]. The application of proteomic techniques to diseases is known as clinical proteomics. This constitutes a powerful tool for studying alterations in pathways implicated in diseases, and identifying novel protein biomarkers of disease. A proteomic biomarker is defined as “a specific peptide or protein that is associated with a specific condition, such as the onset, manifestation, or progression of a disease or a response to treatment” or a “characteristic that is measured as an indicator of normal biological processes, pathogenic processes, or a response to an exposure or intervention” [12].

### 2.1. Qualitative and Quantitative Proteomics

#### 2.1.1. Qualitative Proteomics

**Figure 1:**
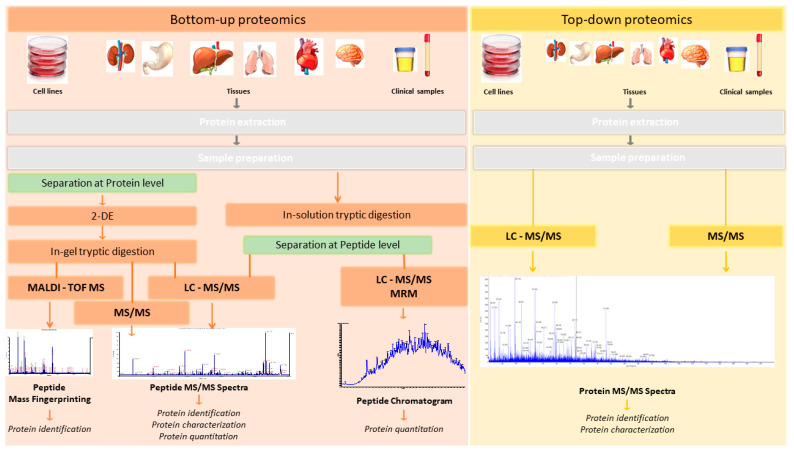
“Bottom-up” and “top-down” Proteomic Strategies in clinical samples. In “bottom-up” proteomics approaches, proteins are processed with an exogenous protease (e.g., trypsin), producing internal peptides that have adequate properties for LC MS/MS and database searches, including length, ionization, and fragmentation parameters. These peptides can be analyzed by shotgun proteomics methods to identify the parent protein based on the endogenous cleavage site, and thereby identify the protein itself. By contrast, top-down proteomics permits both strategies: protein cleavage with proteases and direct protein identification without cleavage.

Two main strategies are used to analyze the proteome: “bottom-up” and “top-down” proteomic strategies [16] (Figure 1).

In the bottom-up approach (shotgun proteomics), the sample is first subjected to separation (by 2D-PAGE or liquid chromatography) and proteolytic digestion (normally using trypsin as the digestion enzyme), and then analyzed by MS/MS. Proteins are identified by matching the masses of proteolytic peptides or their tandem mass spectra with theoretical “in silico” digestions performed using bioinformatics tools applied to protein databases (normally using Uniprot: https://www.uniprot.org/ (accessed on 1 October 2022). Bottom-up proteomics is the most widely used approach for protein identification and characterization. The enzymatic peptides are usually identified by Matrix-assisted laser desorption/ionization-time of flight (MALDI-TOF) or nanoflow/micro liquid chromatography coupled to tandem mass spectrometry analysis (LC-MS/MS). Normally, MS/MS analysis is performed in data-dependent acquisition (DDA) (described in the following section) mode, which generates a unique pattern of fragment masses following fragmentation of a single peptide [17]. However, there are several limitations to bottom-up proteomics approaches. First, the percentage coverage of the protein sequence is low, as only a small and variable fraction of the total peptide population of a protein can be recovered. Genomic studies have shown that each open reading frame can give rise to distinct protein isoforms, owing to alternative splicing products and differences in cell location and post-translational modification (PTM). Limited sequence coverage means that much information about PTMs and alternative splice variants is lost.

While proteomics techniques such as 2D-PAGE gels and mass spectrometry by MALDI-TOF ionization were widely used in the past, their use has waned owing to their inherent limitations [16,18,19,20]. As shown in Figure 1, these correspond to bottom-up proteomics approaches.

**Figure 2:**
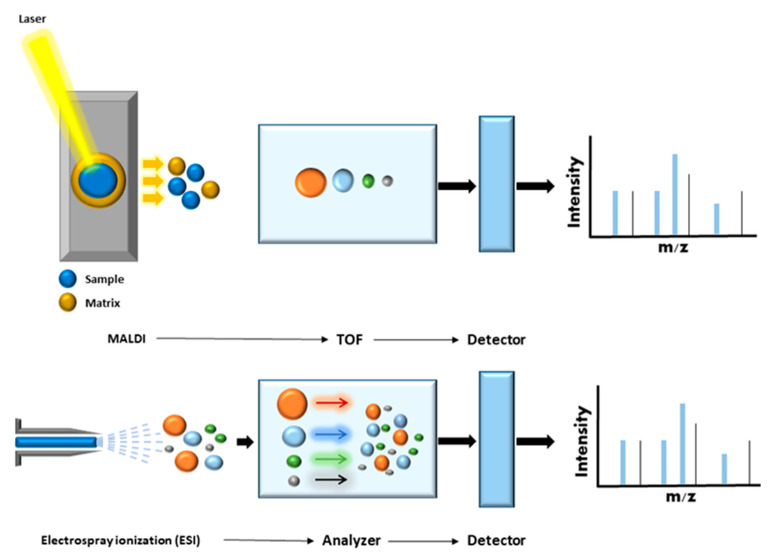
MALDI and ESI Ionization. In MALDI ionization, samples are co-crystallized with an organic matrix on a MALDI plate. A pulsed laser irradiates the co-crystals. This induces rapid heating and desorption of ions into the gas phase. Ions pass through the mass analyzer, such that the smaller peptides reach the detector before the larger peptides (time of flight; TOF). The detector registers the numbers of ions at each individual mass-to-charge (m/z) value, and then the peptide mass fingerprint is generated. In ESI-MS, sample molecules (peptides) are ionized directly in solution. The peptide solution passes through a heated capillary device, and droplets of solution are then sprayed into a vacuum chamber containing a high-strength electric field. The resulting ions pass through a mass analyzer and reach the detector, generating complex spectra with multiply charged ions.

MS/MS was developed at the end of the 19th century in order to measure atomic mass, and was used to demonstrate the existence of isotopes at the beginning of the 20th century [21]. MS is an analytical approach that can measure the mass-to-charge ratio (m/z) of chemical compounds and then calculate their exact molecular weight. Different ionization techniques are used to analyze chemical structures in biological systems. The most widely used are laser desorption/ionization (LDI), matrix-assisted laser desorption ionization (MALDI), surface-enhanced laser desorption/ionization (SELDI), and electrospray ionization (ESI) [22,23]. In the late 1980s, soft ionization (MALDI and ESI) was applied to protein analysis, heralding a revolution in protein analysis. In MALDI ionization, the analyte is embedded in a typically acidic matrix that can absorb large quantities of UV light. This matrix is then excited using a short laser pulse and parts of this matrix are rapidly heated and vaporized/ionized together with the analyte [23]. In ESI ionization, an electric field is applied to the analyte solution flowing through a capillary connected to a liquid chromatographic system. At the end of the fine tip of the capillary is a counter electrode. The solvent is evaporated, and the droplet size decreases, producing smaller droplets and finally yielding charged ions that enter the mass spectrometry system. Both techniques have been widely used in clinical proteomics [14] (Figure 2).

MALDI mass spectrometry has also advanced considerably. In MALDI imaging, the use of a matrix allows capture of the spatial component of a sample. This technique can also be combined with complex image analysis software for biomedical science applications. Indeed, MALDI mass spectrometry has become widely used for rapid and accurate identification of bacteria, mycobacteria, and certain fungal pathogens in clinical microbiology laboratories [18].

Electrospray ionization enables continuous ion generation and can easily be combined with liquid chromatography and various mass analyzers. Recent advances in ESI sources have greatly improved sensitivity, and this technique is now the most commonly used in large-scale qualitative and quantitative proteomics. In the present review, we focus on the most recent proteomics technologies, which combine liquid chromatography and electrospray ionization tandem MS (LC-ESI MS/MS or LC-MS/MS) and represent the most effective and commonly used approaches for proteome analysis [8,11,16], as well as current and future MS-based proteomics strategies for the study of rare genetic and metabolic disorders.

In top-down approaches, no proteolytic enzymes are used and the protein analysis is performed on intact proteins. The procedure includes protein separation, mass spectrometry detection, and data analysis stages. This method can be used to characterize intact proteins from complex biological systems. In contrast to bottom-up approaches, top-down methods provide almost 100% sequence coverage and complete proteoform characterization. In summary, the main advantages of top-down strategies are the ability to (i) generate a complete protein sequence; (ii) locate and characterize PTMs; and (iii) determine protein isoforms. However, certain disadvantages should also be borne in mind, including the high associated cost and the low dissociation efficiency associated with some of these techniques (ECT, ETD), requiring long ion accumulation, activation, and detection times.

#### 2.1.2. Quantitative Proteomics Approach

The presence/absence and overexpression/down-regulation of certain proteins can provide crucial information to help fully characterize the proteomes of cells or tissues. The greatest challenge is to understand how and why protein levels differ between one condition and another, and between healthy versus diseased states.

**Figure 3:**
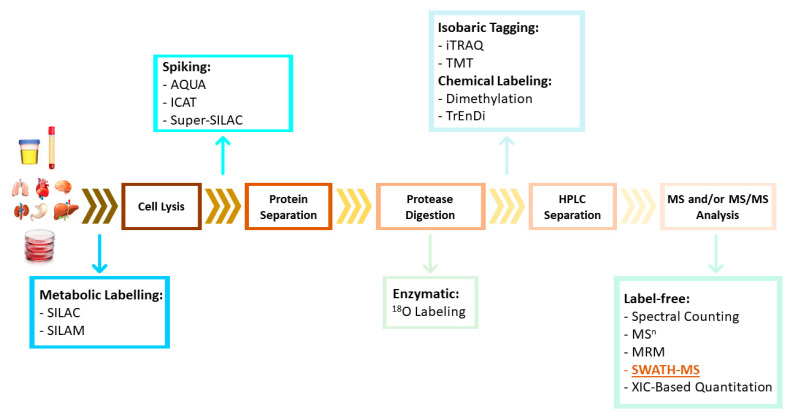
Quantitative methods for proteomics. A quantitative proteomics workflow involves protein extraction/preparation and digestion followed by LC-MS/MS analysis; therefore, there are multiple time points at which peptide quantitation strategies may be introduced. They can be included in cell culture or animal model samples that can be labeled metabolically at the protein level. After cell lysis or protein purification (in clinical samples), labeled proteins can be spiked. During enzymatic digestion, enzymatic labeling can be performed using ^18^O. After digestion, peptides can be labeled chemically or isobarically. Finally, label-free techniques can be used: in these cases labeling of samples is not required, and quantitation is performed during or after data analysis.

MS-based quantitative proteomic approaches can be divided into two general categories: label-based and label-free techniques. Label-based approaches include protein/peptide chemical or enzymatic labelling (e.g., two-dimensional difference in-gel electrophoresis [2D-DIGE], isobaric tag for relative and absolute quantitation [iTRAQ], tandem mass tags [TMT], and 18O-labeling), and metabolic labelling (e.g., stable isotope labelling by amino acids in cell culture [SILAC] and isotope-coded affinity tag [ICAT]) [24,25] (Figure 3). These methods are based on the incorporation of stable isotope labels within peptide/proteins. Heavy and light tags can be added to proteins in different samples (without affecting protein and/or peptide properties such as retention time on a chromatography column) and can be detected by MS to reveal differences in protein expression based on the abundance of each tag type. However, these methods for proteomic quantification are usually more costly (labeling kits are expensive), require additional sample preparation steps, and have limited multiplicity [25].

Recent advances in these techniques include the emergence of label-free approaches, which avoid the inconveniences associated with labelling and enable high-throughput and large-scale protein analysis. Label-free quantitative approaches are based on the comparison of different features between independent LC-MS or LC-MS/MS measurements. The most commonly used label-free methods can be divided into two groups: (i) spectral counting (SC), which involves counting the number of identified peptides or acquired fragment spectra; and (ii) methods that compare precursors’ ion intensities, as determined based on the extracted ion chromatogram (XIC) [25].

**Figure 4:**
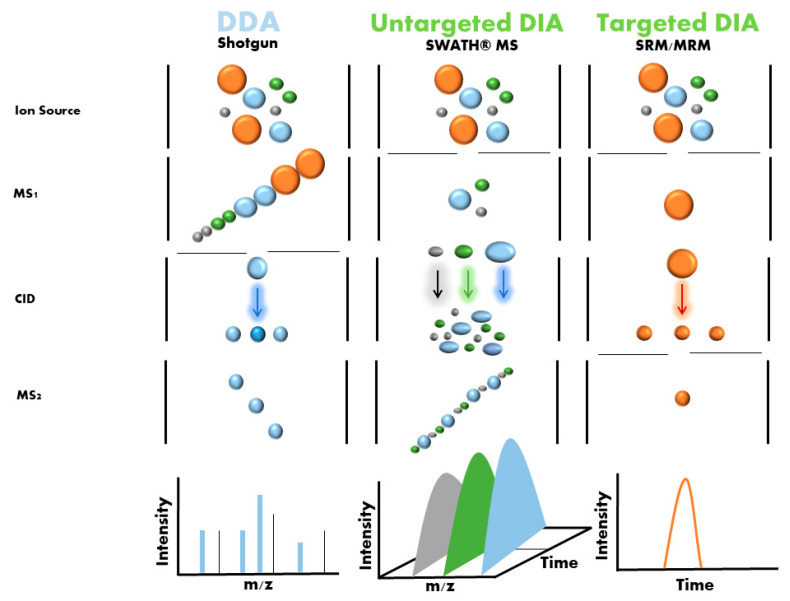
DDA and DIA mode in proteomic analysis. In DDA mode, extracted proteins are digested and directly analyzed by single-shot DDA. The acquired data is searched against a database of known protein sequences and further processed using diverse software tools. In DIA mode, two distinct forms of proteomic analysis can be performed: SWATH-MS (untargeted DIA) and SRM/MRM (targeted DIA). In SWATH-MS extracted proteins are digested and directly analyzed by single-shot analysis in order to generate the spectral library. Once the library is generated, a multi-window running method is applied to individual samples; wide isolation windows span the entire MS1 m/z range and all precursor ions in the library that are found in each isolation window are fragmented in MS2. All proteins found in the library are quantified (untargeted) and all samples are processed using data analysis software to obtain the quantitative data. In MRM and SRM, the precursor ions to be analyzed are fixed (targeted) by the user, and only these are fragmented in MS2.

One emerging label-free strategy is sequential window acquisition of all theoretical mass spectra (SWATH-MS), which was first described by Gillet et al. in 2012 [26]. In this method, non-labeled protein samples are digested using trypsin, and the resulting peptides are analyzed by liquid chromatography coupled with a tandem mass spectrometer (LC-MS/MS) running in an untargeted data-independent acquisition (DIA) mode (Figure 4). In this mode, all ionized peptides that fall within a specified mass range are fragmented in a systematic and unbiased manner using over 100 consecutive, slightly overlapping precursor isolation windows, with a width of 25 m/z each [27]. These overlapping isolation windows are derived from previously generated spectral libraries. These libraries are usually generated using pools and DDA (data-dependent acquisition) of samples from the different groups, although it is now also possible to download regenerated libraries (http://www.swathatlas.org/ (accessed on 2 October 2022)).

#### 2.1.3. Targeted Proteomics

Protein mass spectrometry generally is classified into untargeted (“discovery”) proteomics and targeted proteomics. The aim of untargeted proteomics (the form discussed so far in this paper) is the identification and/or quantification of all proteins (protein discovery). This entails certain limits in terms of precision and accuracy. Thus, it is necessary to carefully standardize MS-based proteomics workflows so that they can be used for the same type of analysis in different laboratories in order to facilitate the discovery of protein biomarkers.

Targeted proteomics is better suited to biomarker candidate verification and clinical applications (protein validation). This approach allows for multiple/selected reaction monitoring mass spectrometry (MRM/SRM)-MS assays to be performed with short run times, has extensive multiplexing capabilities, and offers significantly greater quantitative precision for biomarker validation (Figure 4). This technique also has a high level of reproducibility, which is necessary for use in clinical and preclinical research [28,29].

It should be noted that methods such as MRM/SRM/PRM-MS allow for quantification of absolute protein levels. Other previously described methods such as label-based (e.g., ITRAQ, SILAC) and non-label-based methods (e.g., SWATH) are semi-quantitative methods that only indicate the fold change in protein levels between two conditions.

Targeted MS/MS allows absolute quantification of targeted proteins using isotopically labelled peptide standards (AQUA) [30,31,32] or protein standard absolute quantification (PSAQ) [31,33,34]. Using isotope standards simplifies sample preparation and minimizes variation between mass spectrometry devices, thereby increasing the reliability and accuracy of results.

#### 2.1.4. Affinity-Based (Probes and Antibodies) Proteomics Approaches [35,36,37,38]

Affinity-based proteomics is a relatively new field of proteomics that seeks to characterize protein activity and protein–protein interactions. This method also allows monitoring of the functional regulation of enzymes in complex proteomes. This technology utilizes small molecule activity-based probes (APBs) or protein antibodies. The probe molecules covalently modify the enzyme active site and allow detection and affinity purification of a target enzyme population. Affinity-based antibodies are mainly generated using microarrays or directly by antibody immunoprecipitation of the whole sample. After affinity purification (pulldown based on probes or antibodies), substrates and their exact cleavage sites are identified by mass spectrometry. Moreover, cleavage sites can be quantitatively evaluated by incorporation of quantitative proteomic techniques such as iTRAQ and SILAC [39]. Thus, this technology is highly selective and can be used for analysis of complex samples such as biological fluids, cell lysates, intact cells, and even whole organisms, and can therefore be applied to the study of IMDs.

All activity-based probes share a similar structure [35,39]. They have elements required for targeting, modification, and detection of labeled proteins. Structurally, these elements can be divided into the reactive group, linker, and tag. The reactive functional group provides a covalent attachment to the catalytic residue of the enzyme active site. The linker function separates the reactive functional group from the tag. Finally, the tag enables visualization and/or purification of labeled proteins.

For the antibody microarray, the data set correlates directly with the number of antibodies included in the array and the range of their specificities [37].

Technologies that reveal the most abundant proteins found in certain sample types (e.g., serum) are crucial for the development of biomarkers that can be used as measurable indicators of disease severity and progression, patient stratification, and drug development. Proximity Extension Assay (PEA) is a novel approach based on the interaction between protein antibodies and DNA [38] that has been commercially developed for the analysis of secreted proteins in serum and blood plasma. This technology can translate protein information into actionable knowledge by linking protein-specific antibodies to DNA-encoded tags. It offers high specificity and sensitivity (sub-pg/mL), enabling high multiplex assays with coverage across a broad dynamic range (~9 log), while consuming minimal quantities of sample. In PEA, matched pairs of oligonucleotide-labeled antibodies bind to their target antigens in a pairwise manner. Upon antibody binding, the matched oligonucleotides are brought into proximity and, using a DNA polymerase, a PCR target sequence is created, amplified, detected, and quantified. This downstream process analysis is usually carried out by qPCR or by techniques such as next-generation sequencing (NGS).

The above are all recent technologies, and the coming years will likely see continued growth in the development and application of affinity-based proteomic approaches. The use of probes that target diverse enzyme families has led to a range of applications for activity-based proteomics, in the same way that linking proteins to proteins has provided information about the interactome, and protein-DNA interactions have provided further knowledge about cell processes. Therefore, as in other proteomics approaches, comparison of enzyme activity profiles and protein–protein or protein–DNA interactions (based on antibody precipitation) in healthy versus diseased samples can aid the identification of novel biomarkers and drug targets. The results obtained to date show that affinity-based proteomics offer great promise as imaging tools for disease diagnosis and preclinical and clinical testing of therapeutic agents in vivo.

## 3. Proteomics for the Study of IMD

A key challenge in molecular biology is understanding how changes in the genotype give rise to a given phenotype. Proteins are the final product of genes and the principal effect of molecules in the cell. However, the result of genetic perturbations on the proteome remains poorly studied and understood. Until recently, analysis of the complete proteome was impossible, and therefore studies focused on specific organelles or functions, such as mitochondrial function [40], kinase activity [41], or central carbon metabolism [42]. These studies demonstrated the importance and utility of systematic proteomic analysis to understand both gene function and gene networks. Proteomics can reveal impairment at the level of protein synthesis, stability, degradation, and signaling, all of which can play crucial roles in disease states.

Two main techniques are used to study the proteome: (i) mass spectrometry-based and (ii) antibody-based (e.g., ELISA, Western blot) techniques. The latter offer lower throughput than the former; consequently, MS-based techniques are the first choice for the analysis of complete complex proteomes. In 2019, Grabowski et al. [43] demonstrated that MS-based proteome analysis can be used to guide targeted genetic diagnostics and to uncover the underlying genomic mutations in patients.

**Figure 5:**
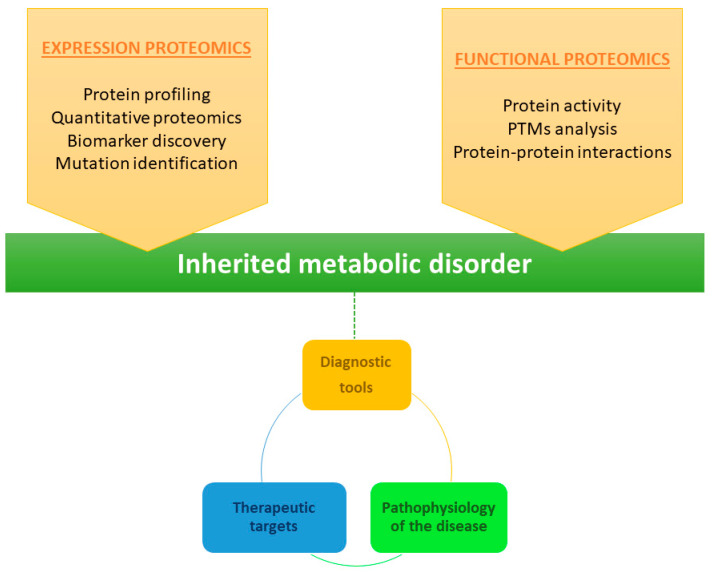
Proteomics methods used for the characterization of inherited metabolic disorders. A combination of expression proteomics (quantitative analysis of protein expression across different samples) and functional proteomics (analysis of the properties of molecular networks in a living cell) can be very useful in the search for diagnostic biomarkers and therapeutic targets in IMDs. Moreover, the information acquired using these two approaches can help to better understand the pathophysiology of the disease.

Proteomics is therefore a powerful approach for identification of proteins implicated in metabolic diseases. In general, proteomics can be divided into three distinct categories: expression proteomics; structural proteomics; and functional proteomics (Figure 5) [44]. Expression proteomics provides information on qualitative and quantitative changes in the proteins present in the proteome. Structural proteomics is used to characterize the protein structure or the protein complexes present in a cell type or organelle. Finally, functional proteomics enables characterization of the biological function and cellular mechanisms of proteins [45]. Combined knowledge of protein function, activity, and post-translational modifications, as well as protein–protein interactions, provides valuable information about cell stage and therefore about the disease. In the study of rare diseases, this information can facilitate the development of more diagnostically useful tools and identification of therapeutic targets, ultimately providing more information about the pathophysiology of the disease [45].

Integration of data acquired using different -omics approaches are essential to fully realize the concept of personalized medicine. Several articles have described the integration of the Exome/Genome Sequence into transcriptomics [46,47], metabolomics [48,49], and proteomics [50] approaches. A variety of tools [51,52,53,54,55] allow the combination and analysis of data derived from different -omics methods. Therefore, including data from several -omics approaches in a systems biology analysis can help improve the diagnostic yield and advance our understanding of the molecular mechanisms underlying the disease.

Plasma samples are typically used for proteomic or other -omics analysis, as plasma can be readily and noninvasively obtained, although disadvantages associated with plasma samples include the presence of abundant proteins (e.g., albumins, immunoglobulins), many of which are glycosylated [56]. Analysis of samples obtained before and after disease onset and during treatment can help identify molecular markers of disease pathogenesis and characterize the effects of treatment. Detection of alterations in proteins obtained from mitochondrial [57], peroxisomal [58], or lysosomal [59] preparations can provide even more valuable information. However, the use of serum samples (which contain many major proteins) can mask differences in mitochondrial, peroxisomal, or liposomal proteins. In diseases in which the kidney is primarily affected, urine samples can be more useful that serum samples in the search for biomarkers.

In recent years, LC-MS/MS techniques and other affinity-based proteomics approaches have been more widely used to obtain more information about rare diseases (Table 1). Here, we summarize some of the most important results obtained using proteomics approaches.

Proteomic changes in methyl malonicaciduria (MMA, OMIM251000) disease have been studied for many years, making this one of the rare diseases that is best studied using proteomic techniques. It is known that methylmalonic acid accumulates toxic metabolites from propionyl-CoA and other compounds upstream of the blockade in the enzyme pathway. Patients develop life-threatening acidosis, respiratory distress, brain disturbance, hyperammonemia, and ketosis. In 2020, Caterino et al. [60] performed a shotgun label-free quantitative proteomics and bioinformatics analysis of HEK 293 cells mutated using the CRISPR/Cas9 technique. Their findings revealed alterations in the cellular architecture and morphology and ROS overproduction in mutated cells. Moreover, they observed alteration of proteins involved in the cytoskeleton and organization of cell adhesion, cell trafficking, mitochondrial, and oxidative processes. The authors generated a cell model of MUT rescue in order to control the specificity of MUT knockout. Proteomics analysis of both models revealed increased susceptibility to propionate- and H_2_O_2_-induced stress through impairment of mitochondrial functionality and imbalances in the oxidation–reduction processes in MUT-KO cells.

Fabry disease (OMIM 301500) is a rare X-linked lysosomal storage disease (LSD) caused by a deficiency in α-galactosidase A. This deficiency leads to the progressive accumulation of glycosphingolipids, mainly globotriaosyl ceramide (Gb3), globotriaosyl sphingosine (lyso-Gb3), and galabiosyl ceramide, and their isoforms/analogues in the vascular endothelium, nerves, cardiomyocytes, renal glomerular podocytes, and biological fluids. Most patients with this disease show no symptoms in the first years of life, and survive into adulthood, but experience permanent and intense pain. These patients can develop early subclinical organ dysfunction from childhood without phenotypic manifestations, resulting in major delays in diagnosis and initiation of enzyme replacement therapy (ERT). One of the main manifestations of this disease is nephropathy; therefore, total urinary protein and albumin excretion is considered one of the most important indicators of this nephropathy.

Some proteomics studies of urine and plasma samples have sought to identify early protein biomarkers and markers of therapeutic response, and while many potential biomarkers have been described, none have yet been used in clinical practice. In 2015, Matafora et al. [64] performed a label-free LC-MS/MS analysis of urine samples in order to assess differences in protein expression between ERT and healthy control (HC) patients. The authors identified several dysregulated proteins, including uromodulin, prostaglandin H2 D-isomerase, and prosaposin. These proteins are implicated in the processes that may contribute to the pathophysiology of Fabry disease, including inflammation and glycosphingolipid metabolism. Furthermore, this study enabled discrimination of female patients from healthy patients; demonstrating the regulation/normalization of some proteins after therapy in these female patients [64]. Using the same proteomics technique, an earlier study of 10 pediatric Fabry patients reported decreased urine levels of prosaposin and GM2 activator protein after 12 months of ERT treatment [65]. Both studies thus confirmed upregulation of prostaglandin H2 D-isomerase and prosaposin expression in urine from Fabry disease patients, and subsequent decreases in these levels after ERT treatment. These findings point to the potential utility of these markers for monitoring the response to ERT. More recently, Doykov et al. [67] performed a similar analysis of urine samples, comparing samples from early-stage/asymptomatic Fabry disease patients with those from healthy controls. The results revealed increased levels of six urinary proteins in the early-stage/asymptomatic Fabry disease group (specifically, albumin, uromodulin, α1-antitrypsin, glycogen phosphorylase brain form, endothelial protein receptor C, and intracellular adhesion molecule 1). The authors concluded that an increase in urine albumin levels may indicate presymptomatic disease. Glycogen phosphorylase brain form was the only protein that was upregulated in early-stage/asymptomatic patients and was further upregulated as multi-organ involvement progressed. Other proteins, such as podocalyxin, fibroblast growth factor 23, cubulin, and alpha-1-microglobulin/bikun in precursor (AMBP), were upregulated only in disease groups, and were specifically associated with kidney disease. Moreover, prosaposin was upregulated in all symptomatic groups, and was associated with disease severity with a high level of specificity [67].

Hollander et al. [66] searched for plasma biomarkers of Anderson–Fabry disease (AFD) using a mass spectrometry iTRAQ proteomic approach (discovery phase) and SRM (validation phase). SRM identified an 8-protein biomarker panel (22 kDa protein, afamin, α-1 antichymotrypsin, apolipoprotein E, β-Ala His dipeptidase, hemoglobinα-2, isoform 1 of sex hormone-binding globulin, and peroxiredoxin 2) that was specific and sensitive to male AFD patients. In female patients, a 9-protein biomarker panel was identified. Only three proteins (apolipoprotein E, hemoglobin α-2, and peroxiredoxin 2) were common to both sexes, again suggesting the existence of a sex-specific alteration in plasma biomarkers in AFD patients [66]. In fact, females develop clinically significant disease.

Gaucher disease (GD, OMIM 230800) is another LSD caused by mutations in the glucocerebrosidase (GBA) gene. This is the most common genetic risk factor for Parkinson’s disease. We have found only one study of GD using a modern proteomics technique (quantitative I-TRAQ assay), that of Pawliński et al. (2020) [68]. This observational, cross-sectional analysis included twelve adults: six GD patients and six healthy controls. Over 400 proteins were analyzed in fasting venous blood samples. The authors sought to determine whether significant differences in protein concentrations between patients and controls were correlated with corresponding changes in gene expression. They identified 31 proteins that differed significantly between the patients and controls. The majority were implicated in regulation of inflammation and hemostasis. Expression of proteins involved in inflammation, including alpha-1-acid glycoprotein 2, S100-A8/A9, adenylcyclase-associated protein 1, haptoglobin, and translationally controlled tumor protein (TCTP) related to inflammation process, was significantly increased in patients versus controls, whereas other proteins implicated in the humoral response (IGHM and IGHG4, and complement C3/C4 complex) were significantly decreased in patients versus controls [68].

Mucopolysaccharidosis (OMIM 252940, 253000, 253010, 253200, 253220) is perhaps one of the most studied of all LSDs. Mucopolysaccharidosis is caused by a deficiency in enzymes required for the stepwise breakdown of glycosaminoglycans. In the preceding decades, the enzyme deficiencies associated with each disease have been identified, leading to the characterization of sevem distinct forms of mucopolysaccharidosis. Several of these are described below, as well as some of the most interesting proteomic analyses of this group of LSDs.

Based on symptom severity, mucopolysaccharidosis type I can be divided into three clinical categories: Hurler and Scheie syndromes, which correspond to phenotypes at either extreme of the clinical spectrum; and Hurler–Scheie syndrome, which corresponds to an intermediate-severity phenotype. All are caused by a deficiency of inalpha-L-iduronidase, which leads to multisystemic accumulation of the glycosaminoglycans (GAGs) heparan and dermatan sulphate. Distinction between the different forms of this disease is based only on clinical criteria, including the rate of symptom progression. Patients with the severe form (Hurler syndrome) develop mental retardation and cognitive decline from the first years of life. Patients with the attenuated forms (Hurler–Scheie and Scheie syndromes) show no brain abnormalities but experience mild-to-moderate impairment in cognitive function. In their 2015 proteomic analysis, Baldo et al. [69] compared the proteome of normal and MPS I mouse hippocampus in order to identify proteins implicated in the brain dysfunction characteristics of this mouse model. Shotgun proteomics analysis by LC-MS/MS identified 297 proteins, of which 32 were differentially expressed. Cathepsins B and D and glial fibrillary acid protein (GFAP) were markedly upregulated in MPS I mouse hippocampus. Using immunohistochemistry techniques, the authors identified the neuroinflammatory process that could be responsible for neuronal dysfunction. No differences in the expression of ubiquitin or other proteins related to protein folding were observed, suggesting no dysfunction of the ubiquitin system. The authors also observed alterations in several proteins involved in synaptic plasticity, including overexpression of post synaptic density-95 (PSD95) protein and decreased expression of microtubule-associated proteins 1A and 1B. These findings suggest that the cognitive impairment in MPS I mice is not due to massive cell death, but rather to neuronal dysfunction caused by neuroinflammation and alterations in synaptic plasticity [69].

In 2020, van den Broek et al. [71] investigated systemic inflammation in untreated MPS I patients and evaluated the effect of HCT (hematopoietic cell transplantation) on systemic inflammation. The authors used dried blood spot samples from patients with HCT-treated MPS using a OLINK Proseek Multiplex inflammation platform. In total, 92 markers of the OLINK inflammation panel were measured in MPS patients compared with those of age-matched control subjects. The authors observed normal leukocyte enzyme activity levels in 93% of patients post-HCT. Moreover, pretransplant samples showed clear differences between patients and controls. The protein markers that distinguished control subjects from patients pre-HCT were mainly pro-inflammatory (50%) or related to bone homeostasis and extracellular matrix degradation (33%). After 10 years of follow-up, only 5 markers (receptor activator of nuclear factor kappa-B ligand, osteoprotegerin, axis inhibition protein 1 [AXIN1], stem cell factor, and Fms-related tyrosine kinase 3 ligand) remained significantly increased, with a large fold-change difference between MPS I patients and control subjects.

To search for markers of MPS disease severity and to monitor effects of existing and emerging treatments, Heywood et al. [70] analyzed urine samples from a small cohort of MPS-I, -II, and -VI patients (n = 12) using label-free quantitative proteomics for the biomarker discovery and MRM for biomarker validation. They identified 53 differentially expressed proteins, including some associated with extracellular matrix organization. In a larger validation cohort of patient samples, targeted multiplexed peptide MRM LC-MS/MS revealed that none of the markers were significantly altered in patients with mild disease relative to the healthy controls. β-galactosidase, a lysosomal protein, was upregulated in all disease groups. Collagen type Iα, fatty-acid-binding-protein 5, nidogen-1, cartilage oligomeric matrix protein, and insulin-like growth factor binding protein 7 were upregulated in severe MPS I and II groups. Moreover, the authors showed that the cartilage oligomeric matrix protein, insulin-like growth factor binding protein 7, and β-galactosidase could be used to distinguish the severe neurological form of MPS-II from the milder non-neurological form. Interestingly, Heg1 was the only protein with significantly increased expression in MPS-VI. In 2022, Zang et al. [72] used a combination of immunocapture and MS-based proteomics (Immuno-SRM) in order to quantify GAA and IDUA proteins in dried blood spot (DBS) and buccal swab samples. Absence of these two proteins is associated with infantile onset Pompe disease (IOPD) and severe MPS I, respectively. To validate the technique, the authors assessed the sensitivity, linearity, and reproducibility of protein concentration measurements in healthy control samples. Subsequent analysis in patients allowed quantification of GAA and IDUA with high reproducibility and sensitivity.

Mucopolysaccharidosis IIIB (MPS IIIB) is a metabolic disease caused by a deficiency in the α-N-acetyl glucosaminidase (NAGLU) enzyme, which in turn results in the accumulation of undegraded heparan sulfate. The main clinical manifestations of the disease are profound intellectual disability and neurodegeneration. De Pasquale et al. (2020) [73] used a label-free quantitative proteomics approach to assess differences in the proteome profile in brains of MPS IIIB versus control mice. They identified 130 significantly deregulated proteins, 74 of which were upregulated in MPS IIIB versus wild type (WT) brains. These proteins were mainly involved in cytoskeletal regulation, synaptic vesicle trafficking, and energy metabolism. The findings constitute an important starting point for future studies seeking to identify novel therapeutic targets for MPS IIIB.

Recently, we demonstrated that using a combination of shotgun proteomics with SWATH-MS [74,75,76] can provide useful information about mucopolysaccharidosis type IVA (MPS IVA). Moreover, this technique can be scaled to accommodate thousands of samples without compromising measurement precision. We are also using proteomics analyses to identify new delivery systems to improve conventional ERT treatment by using nanoparticles to facilitate access of the enzyme to affected organs, such as the brain or bones.

MPS IVA (OMIM 253000) is an LSD caused by mutations in the N-acetylgalactosamine-6-sulfatase (GALNS) gene that promotes the accumulation of large quantities of keratin sulphate (KS). Some of the most characteristic symptoms are systemic skeletal dysplasia due to disruption of cartilage and its extracellular matrix, leading to growth imbalance. ERT with alpha elosulfase, a recombinant human GALNS, constitutes the only systemic treatment. However, no correlation has been described between therapeutic efficacy and urine levels of KS, which accumulates in MPS IVA patients. Moreover, this therapy has a limited impact on skeletal dysplasia, as the infused enzyme cannot penetrate cartilage or bone. Therefore, a key unmet need is the development of an alternative therapeutic approach that ensures cartilage penetration. We have developed a new drug delivery system based on a nanostructure lipid carrier with the capacity to immobilize enzymes used for ERT and to target the lysosome [74]. In our first proteomic study using SWATH-MS, we assessed the effect of the encapsulated enzyme using this new delivery system in fibroblasts from anterior forearm tissue. Our improved ERT technology, using nanoparticles, allows us to achieve greater enzyme internalization inside the cells. SWATH-MS confirmed the improvement of cellular protein routes previously impaired by the disease, compared with cells treated using conventional ERT. In 2020, we applied SWATH-MS analysis to leukocyte samples from HCs (n = 6) and both untreated (n = 5) and ERT-treated (n = 8, sampled before and after treatment) MPS IVA patients in order to identify potential disease biomarkers [75]. We identified 690 proteins, from which we selected those that were dysregulated in ERT-treated MPS IVA patients, and identified four potential protein biomarkers, all of which are implicated in bone and cartilage metabolism: lactotransferrin, coronin 1A, neutral alpha-glucosidase AB, and vitronectin. In 2021, we performed another SWATH-MS [76] study to assess the validity of potential serum biomarkers proposed by other authors and to identify new biomarkers. We used plasma samples from healthy controls (n = 6) and untreated (n = 8) and ERT-treated (n = 5, sampled before and after treatment) MPS IVA patients. We detected 215 proteins with altered expression in ERT-treated MPS-IVA patients versus controls, and identified four potential protein biomarkers involved in bone and cartilage metabolism: fetuin-A, vitronectin, alpha-1antitrypsin, and clusterin. The fact that several proteins were identified as candidate biomarkers in both studies strongly supports their use for this purpose, although more studies focusing on cartilage and bone samples from MPS IVA patients will be required in order to verify/validate their utility as biomarkers of MPS IVA.

Cerebral adrenoleukodystrophy (CALD, OMIM 300100) is an inflammatory neurodegenerative disease associated with a mutation in the *ABCD1* gene that causes the buildup of very long chain fatty acids (VLCFAs) in the brain. VLCFA accumulation can destroy the protective myelin sheath around nerve cells. The lifetime prevalence of developing progressive white matter lesions is about 60%. Early identification of CALD is critical, as legions can be prevented using an allogeneic hematopoietic stem cell therapy, but only at early disease stages. In the last 5 years, two proteomic studies of cerebral spinal fluid (CSF) and plasma samples have been performed to identify protein molecular markers that may be related to cerebral demyelination and disease progression.

In 2019, Orchard et al. [77] published a proteomic analysis of CSF samples from young males with active CALD, and described alterations in several proteins, including APOE4, that could constitute candidate markers of inflammation. Moreover, this group assessed APOE4 in 83 young males with CALD prior to hematopoietic cell transplant; therefore, the association of this protein with cerebral disease was identified. Their results showed that compared with non-carriers, APOE4 carriers had a greater burden of cerebral disease, higher gadolinium intensity, inferior neurologic function, and elevated CSF MMP2 levels. These data constitute the first evidence linking APOE4 with increased cerebral disease severity in CALD and suggest that this protein may represent a valid therapeutic target. In 2020, Richmond et al. used blood samples from six well-characterized brother pairs affected by ALD and discordant for the presence of CALD [78] to perform multi-omic profiling, including genome, epigenome, transcriptome, metabolome/lipidome, and proteome analysis. The results revealed discordant genomic alleles in all families, and differentially abundant molecular features across the -omics technologies.

In 2022, Wang et al. [79] used the OLINK Proximity Extension Assay to analyze the cerebral spinal fluid (CSF) of young males with CALD and performed a comparative analysis using plasma samples. Using the Target 96 Neuro Exploratory panel, they found that levels of five proteins were significantly increased in CSF. Only for neurofilament light chain (NfL) was a significant correlation observed between CSF and plasma samples. Young males with CALD showed a 11.3-fold increase in plasma NfL compared with controls. Moreover, nine of eleven young males with CALD who underwent HCT showed a decrease in plasma NfL levels after 1 year of treatment compared with pre-HCT levels. The authors concluded that plasma NfL could constitute a useful determinant of outcomes in CALD.

Phenylketonuria (PKU, OMIM 261600) is a rare genetic disease caused by a deficiency in phenylalanine hydroxylase (PAH) in the liver. This defect induces increases in blood phenylalanine (Phe) and neurotoxicity. Therapies for this disease are mainly based on the restriction of Phe intake, which largely requires modification of the diet. A new therapy based on pegylated phenylalanine ammonia lyase (PEG-PAL) was approved in 2019 by EMA and acts to metabolize Phe into cinnamic acid. Another potential therapy (currently in clinical trials Phase 1/2) consists of a single delivery of a rAAV carrying the PAH gene directly into liver to convert Phe into tyrosine, mimicking the normal process of Phe metabolism. In 2021, Manek et al. [80] performed transcriptomic and proteomic analyses in order to compare these two Phe-lowering strategies. They found that both approaches lower brain Phe and increase neurotransmitter levels, thereby correcting animal behavior. In a more concrete way, these groups investigated the effect of high levels of Phe and how these levels can be lowered in liver functions in PKU mice. The authors observed increased levels of Cyp4a10/14 proteins, which are involved in lipid metabolism, and upregulation of genes involved in cholesterol biosynthesis.

Cystinuria (OMIM 220100) is a rare genetic disease that is mainly characterized by impaired transport of cystine and dibasic amino acids in the proximal renal tubule. About half of all cases are due to a mutation in *SLC3A1* that is inherited in an autosomal recessive manner. The *SLC3A1* gene encodes rBAT, one of the two most important proteins that are in charge of neutral and basic amino acid transport. The other 50% of cases are caused by autosomal dominant *SLC7A9* mutations with incomplete penetrance; *SLC7A9* encodes a protein that is also involved in transport of amino acids. Cystinuria patients often have kidney stones due to the low solubility of cystine in urine. In these patients (cystinuria patients), even though cystine stones were low, morbidity is high due to a high rate of recurrence, the need for multiple surgeries, and the risk of developing chronic kidney disease. Renal function in patients with cystine kidney stones is reduced compared to those with non-cystine stones, but the cause of impaired kidney function is unclear, since gene mutations cannot fully explain kidney stone formation. Therefore, there are likely other factors that underlie this decrease in kidney function, for example proteins that serve as promoters or inhibitors of cystine precipitation, aggregation or epithelial adherence. Further proteomics studies will be necessary to identify these mechanisms. Assinos et al. (2019) [82] conducted proteomic analyses of urine samples to assess differences in the concentration and function of urinary proteins between cystinuria and renal stones in (CYS)patients and healthy controls (HC). They concluded that CYS patients show alterations in urine proteins involved in cellular processes including stress, inflammation, and the immune response.

An earlier study by Bourderiox et al. (2015) studied cystinuria through proteomic analysis of urinary extracellular vesicles in cystinuria patients versus controls [83], identifying 165 dysregulated proteins, 38 of which were upregulated in patients versus controls. The majority of these proteins were markers of kidney injury, circulating proteins, and neutrophil-derived proteins. The authors observed a correlation between specific clusters of urinary proteins and the severity of renal disease, suggesting a possible role of inflammation in kidney disease progression.

Kovacevic et al. (2015) [84] compared urinary proteomes of two pediatric CYS patients with two age- and sex-matched controls, using label-free proteomic analysis. The CYS patients showed altered urinary excretion of proteins associated with oxidative stress and inflammation, leading to fibrosis. However, this study suffered from certain limitations, including a limited number of patients. In 2019, the same group [85] published a proteomics analysis in a long patient cohort to assess differences in the concentration and function of urinary proteins between patients with cystine stones (CYS) and healthy controls. The results again demonstrate proteomic differences between patients and controls. This study identified a large number of proteins (2097), of which 398 were dysregulated between the CYS and HC groups. Of these, 191 were involved in transport processes and 61 in inflammatory responses. A large proportion was vesicle-mediated transport proteins. Of the downregulated proteins, twelve were involved in endosomal transport and nine in transmembrane transport. The more interesting proteins were myosin-2 and 2actin-related proteins that were significantly upregulated in the vesicle-mediated transport group. Taken together, these proteomic findings provide evidence of impaired endocytosis, dysregulation of actin and myosin cytoskeleton, and inflammation in CYS.

Niemann–Pick type C disease (NPC, OMIM607625) is a rare neurodegenerative disorder caused by mutations in the NPC intracellular cholesterol transporter 1 or 2 (Npc1 or Npc2) and is characterized by hypomyelination in the central nervous system (CNS). While Npc1 has key roles in both neurons and oligodendrocytes during myelination, the link between altered cholesterol transport and inhibited myelination is unclear. To better understand this disease, Yang et al. (2019) [86] performed a quantitative mass spectrometry (MS)-based proteomics analysis to compare the protein composition of corpus callosum in WT versus NPC mice. They identified a total of 3009 proteins, including myelin structural proteins, neuronal proteins, and astrocyte-specific proteins. Moreover, they observed downregulation of myelin structural and indispensable proteins in Npc1 mutant mice. The observed reduction in ceramide synthase 2 (Cers2), UDP glycosyltransferase 8 (Ugt8), and glycolipid transfer protein (Gltp) expression, suggesting a potentially altered sphingolipid metabolism in NPC, and possible involvement of Gltp in myelination.

Lesch–Nyhan disease (LND, OMIM 300322.) is an inherited disorder caused by pathogenic variants in the *HPRT1* gene. This gene encodes the purine recycling enzyme hypoxanthine–guanine phosphoribosyltransferase (HGprt). Sutcliffe et al. (2021) [87] performed proteomic and genomic analyses in pluripotent stem cell (iPSC) lines generated from fibroblasts from LND patients and controls. First, the authors fully characterized this iPSC line by immunostaining to detect pluripotency markers. While a gene expression profile generated using RNAseq revealed heterogeneity among LND cells lines, several abnormalities were detected in all LND lines, including reduced mRNA expression ofHPRT1 and FAR2P1 and increased mRNA expression of RNF39. Shotgun proteomics revealed expected abnormalities in the LND lines, including the absence of the HGPRT protein. However, this approach also revealed unexpected abnormalities in LND lines, including increased and decreased expression of GNAO1 and NSE4A, respectively. Using the data obtained from both -omics techniques, the authors demonstrated a good (albeit partial) correlation between the abnormalities detected using RNAseq and proteomics methods.

Mutations in nuclear genes that encode mitochondrial proteins such as very long-chain acyl-CoA dehydrogenase (VLCAD) and trifunctional protein (TFP) cause rare autosomal recessive disorders. VLCAD (OMIM 201475) and TFP (OMIM 609015) deficiency can induce hypoglycemia, cardiomyopathy, intermittent muscle breakdown (rhabdomyolysis), and liver failure. Clinical management of these diseases includes a reduction in the dietary intake of long-chain fatty acids (LCFA), fasting avoidance, and a high carbohydrate diet supplemented with medium-chain triglycerides and in some cases carnitine. Despite some advances in detection and clinical management of this condition, affected patients still experience life-long symptoms due to difficulties performing molecular and biochemical characterization of disease phenotypes. In 2021, Ramiro et al. [88] published the results of their quantitative label-free proteomic analysis in fibroblasts derived from patients with three different VLCAD deficiency mutations and three distinct TFP deficiency mutations. They observed decreased expression of the VLCAD- and TFP-associated proteins in cells carrying the VLCAD and TFP mutations, respectively. Moreover, they described decreased mitochondrial respiratory capacity in cells with VLCAD and TFP mutations after glucose removal, and reduced glycogen levels in cells with TFP mutations. Despite these energetic deficiencies, they observed no changes in mitochondrial morphology, distribution, fusion, or fission, as evaluated using confocal or transmission electron microscopy and corroborated by proteomic and antibody-based protein analysis. The proteomic findings revealed upregulation of both mitochondrial respiratory chain proteins and proteins that facilitate the assembly of respiratory complexes in fibroblasts harboring VLCAD mutations and, to a lesser extent, those carrying TFP mutations. In this model, authors found that expression levels of 45 proteins corresponding to the major intracellular antioxidant networks were similar in cells carrying VLCAD or TFP mutations and non-diseased controls, while levels of catalase and glutathione S-transferase theta-1 were downregulated. Taken together, these data suggest that despite metabolic deficits, cells carrying VLCAD or TFP mutations have the necessary cellular and mitochondrial proteomic integrity required to preserve their architecture, support energy production, and protect against oxidative stress.

Short-chain acyl-CoA dehydrogenase deficiency (SCADD, OMIM 606885) is another rare inherited autosomal recessive disorder, the pathophysiology of which is poorly understood. This disease is caused by genetic variation in *ACADS*, which encodes the enzyme SCAD, a component of the mitochondrial fatty acid oxidation (FAO) system. This enzyme is necessary for the dehydrogenation of butyryl-CoA, the first step in the breakdown of short-chain fatty acids through β-oxidation. Symptomatic SCADD patients exhibit hypotonia, hypoglycemia, lethargy, dysmorphic features, developmental delay, and epilepsy. A 2014 proteomic analysis using quantitative label-free LC-MS/MS [89] characterized the mitochondrial proteome profile of symptomatic patients homozygous for missense variations in *ACADS* in order to link protein alterations to the disease. The analysis identified and quantified some 300 mitochondrial proteins. In patients carrying the c.319C > T variant in *ACADS,* significant alterations with respect to controls were observed for 13 proteins, all with roles in the antioxidant system and amino acid metabolism. In patients carrying the c.1138C > T variant, 22 proteins were significantly altered relative to controls. These proteins were involved in fatty acid β-oxidation, amino acid metabolism, and protein quality control. Finally, expression of three proteins was significantly altered in both patient groups compared with controls: adenylate kinase 4 (AK4), nucleoside diphosphate kinase A (NME1), and aldehyde dehydrogenase family 4 member A1 (ALDH4A1). Two of these are particularly interesting (AK4 and NME1), given their roles in energy reprogramming and cell survival and proliferation, all of which are important pathways in SCADD and related metabolic diseases.

Duchenne muscular dystrophy (DMD; OMIM310200) is a rare X-linked genetic disease with an incidence rate of 1:5000. DMD is caused by frame-disrupting mutations in the gene encoding dystrophin, resulting in loss of dystrophin [90]. Affected boys typically present clinical signs in the first few years of life, with features suggestive of muscle weakness and often with global developmental delay. Progressive muscle weakness leads to loss of ambulation by the age of 10, and if untreated, to fatal cardiorespiratory insufficiency by late adolescence. Establishing a correct diagnosis in dystrophinopathies requires a multidisciplinary approach involving pediatricians, geneticists, and neurologists to define the severity of the clinical phenotype by means of genetic, enzymatic, and immunohistochemical tests [90]. The application of proteomics to the study of dystrophinopathies could facilitate both diagnosis and treatment. In 2014, Ayoglu et al. [36] explored the possibility of identifying circulating candidate protein markers in rare diseases by applying an affinity-based proteomics approach to generate a proteomic profile in blood samples from muscular dystrophy patients versus controls. The authors used an antibody bead array platform with 384 antibodies. Based on the results obtained from analysis of both serum and plasma, they identified eleven proteins associated with muscular dystrophy, of which four were upregulated in blood from muscular dystrophy patients: carbonic anhydrase III (CA3), myosin light chain 3 (MYL3), mitochondrial malate dehydrogenase 2 (MDH2), and electron transfer flavoprotein A (ETFA). These interesting data could aid the development of new clinical tools for management of dystrophinopathies.

## 4. Conclusions

The findings discussed here leave no doubt that proteomics techniques have greatly benefitted the study of rare diseases and have important implications for clinical practice, facilitating the development of more effective diagnostic and prognostic methods, the identification of new therapeutic targets, and the tailoring of individualized patient therapy. While the utility of proteomics for biomarker discovery has been demonstrated, further research is required to enhance performance and reproducibility between laboratories; issues such as pre-analytical variables, analytical variability, and biological sample variation must be addressed before proteomics tools can be incorporated into routine clinical laboratory practice. Moreover, current proteomic techniques require specialist operators and expensive equipment, further limiting their use in clinical laboratories. Nonetheless, in the near future, proteomics will likely be the method of choice for the identification of biological markers of specific diseases and will in turn facilitate the development of new, more precise biochemical and immunological tests.

## Figures and Tables

**Table 1:** Overview of recent proteomics analyses of rare genetic metabolic disorders.

Disease	Disease Type	Altered Protein or Gene	Sample Type	Technique	Reference
Methyl malonic aciduria (MMA)	Methylmalonicacidemia	Methylmalonyl-CoA mutase (MUT) enzyme	HEK 293 cells mutated using CRISPR/Cas9 technique	Label-free LC-MS/MS	[60]
Fabry disease [61,62,63]	Lysosomal storage disease	α-galactosidase A	Human urine samples	Label-free LC-MS/MS (discovery) SRM (validation)	[64]
			Human urine samples	Label-free LC-MS/MS (discovery) SRM (validation)	[65]
			Human plasma samples	iTRAQ labelling (discovery) SRM (validation)	[66]
			Human urine samples	MRM	[67]
Gaucher disease	Lysosomal storage disease	Glucocerebrosidase	Human blood	iTRAQ labelling	[68]
Mucopolysaccharidosis MPS I	Lysosomal storage disease	α-L-iduronidase	Mouse brain	Label-free LC-MS/MS	[69]
			Human urine samples	Label-free LC-MS/MS (discovery) SRM (validation)	[70]
			Dried blood spots (DBS)	OLINK Proseek Multiplex Inflammation	[71]
Mucopolysaccharidosis MPS II	Lysosomal storage disease	Iduronatesulfatase	Human urine samples	Label-free LC-MS/MS (discovery) SRM (validation)	[70]
			Dried blood spots (DBS) and buccal swabs	Immunocapture and LC-MS/MS (Immuno-SRM)	[72]
			Human urine samples	Label-free LC-MS/MS (discovery) SRM (validation)	[70]
Mucopolysaccharidosis MPS IIIB	Lysosomal storage disease	α-N-Acetylglucosaminidase	Mouse brain	LC-MS/MS	[73]
Mucopolysaccharidosis MPIV A	Lysosomal storage disease	N-acetylgalactosamine-6-sulfate sulfatase (GALN)	Human urine samples	Label-free LC-MS/MS (discovery) SRM (validation)	[70]
			Primary fibroblast culture	Label-free LC-MS/MS SWATH-MS	[74]
			Human leukocytes	Label-free LC-MS/MS SWATH-MS	[75]
			Human plasma	Label-free LC-MS/MS SWATH-MS	[76]
Adrenoleukodystrophy (X-ALD)	Peroxisomal disorder	Long-chain fatty acid accumulation in plasma and tissues	CSF samples	iTRAQ labelling	[77]
			Serum samples	Multi-omic approach	[78]
			CSF samples	OLINK Proximity Extension Assay	[79]
Phenylketonuria (PKU)	Amino acid metabolism	Phenylalanine hydroxylase	Mouse liver samples	Label-free LC-MS/MS spectral count	[80]
Cystinuria [81]	Inborn errors of metabolism	Variants in genes *SLC3A1* (type I) *SLC7A9* (type II and type III)	Human urine samples	LC-MS/MS TMT labelling	[82]
			Human exosomes urine samples	Label-free LC-MS/MS	[83]
			Human urine samples	Label-free LC-MS/MS spectral count	[84]
			Human urine samples	LC-MS/MS TMT labelling	[85]
Niemann-Pick type C disease	Cholesterol metabolism	HE1	Mouse corpus callosum	Label-free LC-MS/MS	[86]
Lesch-Nyhandisease	Purine metabolism	Hypoxanthine-guanine phosphoribosyl transferase	Induced pluripotent stem cell (iPSC) lines from fibroblast	Label-free LC-MS/MS	[87]
VLCAD deficiency disease	Fatty acid oxidation disorder	Variants in *VLCAD*	Human primary cell lines with *VLCAD* and *TFP* mutations	Label-free LC-MS/MS	[88]
SCAD deficiency disease	Fatty acid oxidation disorder	Variants in *SCAD*	Primary fibroblasts culture	Label-free LC-MS/MS	[89]
Duchenne muscular dystrophy (DMD)	Dystrophinopathies	Dystrophin	Human blood samples (plasma and serum)	Antibody suspension bead arrays	[36]

## Data Availability

Not applicable.
